# Hodge decomposition of wall shear stress vector fields characterizing biological flows

**DOI:** 10.1098/rsos.181970

**Published:** 2019-02-06

**Authors:** Faniry H. Razafindrazaka, Pavlo Yevtushenko, Konstantin Poelke, Konrad Polthier, Leonid Goubergrits

**Affiliations:** 1 Freie Universität, Berlin, Germany; 2 Institute for Imaging Science and Computational Modelling in Cardiovascular Medicine, Charité-Universitätsmedizin, Berlin, Germany

**Keywords:** wall shear stress, Hodge decomposition, vector field analysis

## Abstract

A discrete boundary-sensitive Hodge decomposition is proposed as a central tool for the analysis of wall shear stress (WSS) vector fields in aortic blood flows. The method is based on novel results for the smooth and discrete Hodge–Morrey–Friedrichs decomposition on manifolds with boundary and subdivides the WSS vector field into five components: gradient (curl-free), co-gradient (divergence-free) and three harmonic fields induced from the boundary, which are called the centre, Neumann and Dirichlet fields. First, an analysis of WSS in several simulated simplified phantom geometries (duct and idealized aorta) was performed in order to understand the nature of the five components. It was shown that the decomposition is able to distinguish harmonic blood flow arising from the inlet from harmonic circulations induced by the interior topology of the geometry. Finally, a comparative analysis of 11 patients with coarctation of the aorta (CoA) before and after treatment as well as 10 control patients was done. The study shows a significant difference between the CoA patients before and after the treatment, and the healthy controls. This means a global difference between aortic shapes of diseased and healthy subjects, thus leading to a new type of WSS-based analysis and classification of pathological and physiological blood flow.

## 1. Introduction

Biological flows or haemodynamics of the cardiovascular system play an important role in the genesis, progress and treatment of cardiovascular pathologies including congenital or acquired diseases of the heart, heart valves and vessels. This is because wall remodelling including wall thickness and wall constitution is triggered by haemodynamics. The major haemodynamic parameter describing an interaction between haemodynamics and a vessel wall, which is covered by endothelial cells, is the wall shear stress (WSS). The WSS is an area-normalized tangential force component of the blood flow acting on the wall and/or endothelial cells. In turn, endothelial cells trigger and modulate adaptation, inflammation and remodelling of the vessel wall as well as a respective remodelling of the vessel lumen [1,2]. Consequently, abnormal WSS is considered an important local risk factor for a set of diseases or pathological processes. These include, for example, atherosclerosis of carotid arteries [3] or coronary artery disease [4], rupture risk of cerebral aneurysms [5,6] or abdominal aortic aneurysms [7], aortic dilatation [8] and thrombus formation [9]. Furthermore, the analysis of WSS is also of great interest for the study of the haemodynamic impact of a treatment or a change of the haemodynamics caused by a certain treatment device. These studies include, for example, an analysis of post-treatment flow conditions after a treatment of cerebral aneurysms with a flow diverter [10] or a change of flow conditions after an aortic valve replacement [11]. The use of WSS as a reliable biomedical marker to characterize disease, disease progress or initiation or to characterize haemodynamic outcome of a treatment procedure is challenging. The WSS is also a surface-bounded vector field with vector magnitudes and directions varying in space and time. This allows for a definition of a set of parameters, which were proposed during the last years as haemodynamic risk parameters for endothelial dysfunction and related wall remodelling. A characterization of WSS magnitude, direction, time and space gradients as well as topological features results in a relatively large set of parameters, which are well summarized in [12,13]. The majority of studies investigating WSS in biological flows are numerical studies using an image-based computational fluid dynamics approach [14], or 4D VEC MRI-based assessment [15]. The primary source of data for the WSS analysis, however, is computational fluid dynamics (CFD), since an accurate WSS assessment requires a high spatial resolution as shown by mesh independence studies for CFD solutions [16].

Vector fields modelling fluid flow often tend to exhibit a complicated behaviour on various scales and are hard to understand. This poses a particular problem for clinical applications where the behaviour of blood flow in vessels serves as an indicator for potential abnormalities. The classical Helmholtz decomposition was a first step to classify and analyse vector fields by decomposing them into a divergence-free component and a component having a potential. With the advent of Hodge theory, Helmholtz’ results generalize to decomposition rules for differential forms on closed manifolds in arbitrary dimensions. Since then a tremendous amount of research—both on the theoretical and on the applied side—has been carried out to include manifolds with boundary, differential forms of Sobolev class and various flavours of Hodge-type decomposition statements, see e.g. [17] for an overview of Hodge-type decompositions and the survey [18].

An important landmark in this evolution is the *L*^2^-orthogonal decomposition of *k*-forms on manifolds with boundary as

$$\Omega^{k}=\text{d}\Omega_{D}^{k-1}⊕\delta \Omega_{N}^{k+1}⊕\text{d}\Omega^{k-1}\cap \delta \Omega^{k+1}⊕(H_{N}^{k}+H_{D}^{k}),$$

where the spaces $H_{N}^{k}$ and $H_{D}^{k}$ of harmonic Neumann and Dirichlet fields, respectively, reflect the absolute and relative cohomology of the manifold. Specifically for vector fields, the first two spaces in this decomposition correspond to divergent and rotational irregularities in the interior of the geometry, whereas the latter three spaces represent steady flows through the domain, as each field in these spaces is harmonic. A fairly recent result [19] provides a further orthogonal decomposition of these spaces into subspaces

$$H_{N}^{k}=H_{N,\mathrm{co}}^{k}⊕H_{N,\partial \mathrm{ex}}^{k}\;\text{and}\;H_{D}^{k}=H_{D,\mathrm{ex}}^{k}⊕H_{D,\partial \mathrm{co}}^{k},$$

which permits a precise distinction between harmonic flows induced by boundary components, represented by the subspaces $H_{N,\mathrm{co}}^{k}$ and $H_{D,\mathrm{ex}}^{k}$, from those induced by the interior topology of the manifold, represented by $H_{N,\partial \mathrm{ex}}^{k}$ and $H_{D,\partial \mathrm{co}}^{k}$.

For the numerical treatment of vector fields, it is therefore important to seek for a discretization which on the one hand provides a good approximation with predictable error, and on the other hand preserves the structural decomposition results from the smooth theory.

In this work, we focus on a discretization by piecewise constant vector fields (PCVF) resulting from CFD-based analyses of the blood flow. PCVFs are a very intuitive and simple to implement approximation while at the same time a concise theoretical framework has been developed in recent years, which includes the aspects of convergence and structural consistency. The recent works [20,21] establish a consistent discretization for PCVFs of the smooth refined decomposition results for vector fields on surfaces with boundary, now including distinguished subspaces for effective boundary analysis and control. Previous to that, a first strategy for the analysis of vector fields is provided by the decomposition in [22], with a convergence analysis on closed surfaces in [23], and a discrete connection for PCVFs is proposed in [24], both without an effective boundary control.

The aim of our study presented here is a proof of concept for the novel Hodge-type decomposition analysis of the WSS vector fields for blood flows in general and specifically for the aortic flow. The paper is structured as follows: first, a theoretical analysis of each vector field component with respect to a WSS vector field is given. Second, a detailed description of the data acquisition and blood flow simulation is exposed. Finally, a statistical analysis of several patients will summarize the results.

## 2. Discrete Hodge-type decomposition

The most important results on discrete Hodge-type decompositions on simplicial meshes concerning our application can be summarized by two fundamental theorems: the traditional Hodge–Helmholtz decomposition decomposes vector fields on closed surfaces into three components. By contrast, on surfaces with boundary a refined decomposition is provided by the so-called Hodge–Morrey–Friedrichs decomposition. The main ingredients of the discretization and the related spaces are given in appendix A. These decompositions constitute the building block of all analysis in the present work. For the theoretical foundations, see [20,21].

**Theorem 2.1 (Hodge–Helmholtz decomposition).**

*The space of piecewise constant vector fields* *Λ*^1^(*M*_*h*_) *on a closed simplicial surface* *M*_*h*_ *decomposes into an* *L*^2^-*orthogonal sum of the spaces of gradient fields, co-gradient fields and harmonic fields*:

$$\begin{array}{rl} \Lambda^{1}(M_{h}) & =\nabla S_{h}⊕J\nabla S_{h}^{*}⊕(H:=\ker\;{\mathrm{curl}}_{h}^{*}\cap \ker\;{\mathrm{div}}_{h})\\ X & =\underset{{\mathrm{curl}}_{h}^{*}\nabla \varphi =0}{\underbrace{\nabla \varphi }}⊕\underset{{\mathrm{div}}_{h}J\nabla g=0}{\underbrace{J\nabla \psi }}⊕\underset{{\mathrm{curl}}_{h}^{*}Y={\mathrm{div}}_{h}Y=0}{\underbrace{Y}}. \end{array}$$

**Figure 1.**
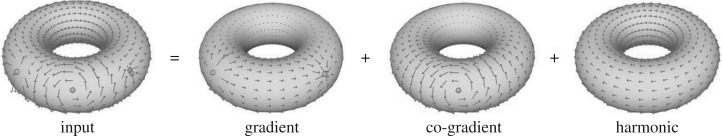
Example of a Hodge–Helmholtz decomposition of a PCVF on a torus into gradient, co-gradient and harmonic field.

The fields belonging to $\nabla S_{h}$ are free of turbulence and contain only flow induced by sources and sinks. J∇ψ is divergence-free and contains the rotational part of the field (figure 1). Furthermore, if *M*_*h*_ is homeomorphic to a sphere with *m* boundaries, then the harmonic fields can be decomposed into three components:

**Theorem 2.2 (Hodge–Morrey–Friedrichs decomposition HMF).**

*On a surface M_h_ homeomorphic to a sphere with m boundaries, the space of harmonic fields can be decomposed into center fields, Neumann fields, and Dirichlet fields*:

2.1 $$\Lambda^{1}(M_{h})=\nabla S_{0}⊕J\nabla S_{0}^{*}⊕\nabla S_{h}\cap J\nabla S_{h}^{*}⊕H_{N}⊕H_{D}$$

**Figure 2.**
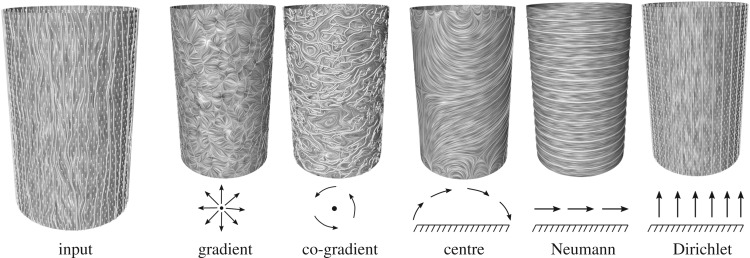
An HMF-decomposition of a perturbed WSS vector field on a cylinder into five components: gradient, co-gradient, centre, Neumann and Dirichlet vector field.

One of the main studies of this paper is to understand the nature of these harmonic spaces on simulated CFD WSS vector fields. Instuitively the space $H_{C}=\nabla S_{h}\cap J\nabla S_{h}^{*}$ of center vector fields behaves similar to the space of smooth vector fields forming an ≈45° angle with the boundaries, the Neumann vector fields are orthogonal to the boundaries, and the Dirichlet are parallel to the boundaries. By the Pythagorian theorem it is

$${∥X∥}^{2}={∥\nabla \varphi \;∥}^{2}+{∥J\nabla \psi \;∥}^{2}+{∥H_{C}∥}^{2}+{∥H_{N}∥}^{2}+{∥H_{D}∥}^{2}$$

which enables a full quantification of the input vector fields according to their decomposition components. Figure 2 shows an example of an HMF-decomposition on the WSS of a simple flow on a cylinder. Notice how the field is dominated by $H_{D}$.

## 3. Wall shear stress component analysis

In this section, we study each component of the HMF-decomposition with respect to the WSS of several phantom as well as real patient models obtained from CFD. The phantom models are either hand-designed or real patient models with mathematical deformation and boundary conditions. The observations are used to emphasize possible changes of WSS encoded in each HMF-component with respect to anatomy/topology of the geometry, and parameters used for blood flow simulation.

### 3.1. Perturbed wall shear stress

**Figure 3.**
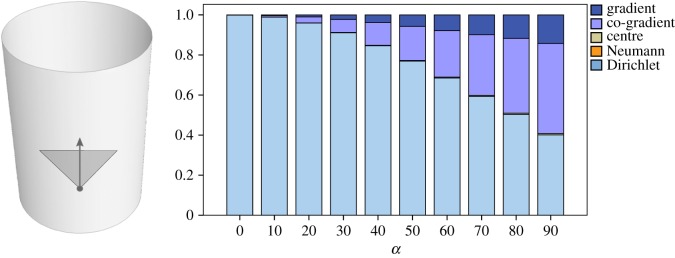
Perturbation of a laminar WSS on a cylinder starting from 0° to 90°. An increase in angle deviation decreases the Dirichlet component and increases the co-gradient components.

Consider a smooth cylinder with a WSS of a laminar flow. We add a moderate amount of rotational noise to the vector field within the interval (− *α*, *α*) where *α* bounds the frequency of the noise. High values of *α* correspond to high overall frequencies while small values alter slightly the global smoothness of the flow. The HMF-decomposition shows that the Dirichlet field $H_{D}$ recovers the original field in its unperturbed state, behaving like a vector field denoising. The increase of *α* decreases $H_{D}$ and increases the co-gradient field. Figure 3 is a quantitative comparison of each decomposition where *α* varies from 0° to 90°. The diagram shows that $H_{D}$ is a good reference to understand the global structure of the WSS.

In general, harmonic fields depend only on the topology of the shape, not the field. In figure 5 (second row), for example, $H_{D}$ stays invariant even though the input velocity profile is changed. Quantitatively half of the WSS component is Dirichlet. One logic behind this is reflected in the nature of fluids, being mostly dominated by a laminar component in order to move only in one direction.

### 3.2. Coarctation analysis

**Figure 4.**
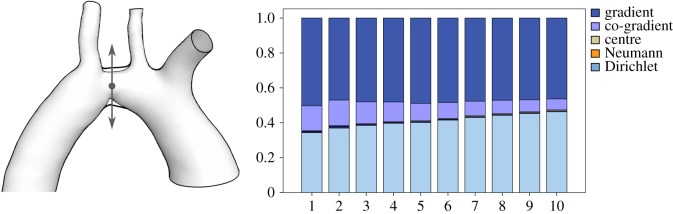
Linear deformation of a pre- to post-intervention of a patient stenosis. A constant input velocity profile is used for the simulation. An increase in the Dirichlet component and a reduction in the co-gradient field is observed within 10 frames of the deformation.

Aortic coarctation is a common congenital heart disease. It represents a local narrowing of the aortic vessel causing abnormal blood flow and pressure in the cardiovascular system. Generally, the WSS vector field of a pre- and post-operative patient does not provide enough information about amelioration in the patient blood flow. The HMF-decomposition enables us in a theoretical setting to identify important changes between the two states. We took a segmented MRI scan of a patient before and after operation, deformed the coarctation linearly from pre to post and analysed the WSS evolution during the diffusion process. The simulation is performed with a plug profile and settings given in §4.1. The results are shown in figure 4. We notice a significant increase in the Dirichlet field amortized with a reduction in co-gradient field. The improvement in the Dirichlet field component corresponds to the improvement of the overall blood flow as proven previously. Notice how the gradient, Neumann and centre fields remain almost unchanged. The nature of these components is explained in the next sections.

### 3.3. Plug versus MRI profile

**Figure 5.**
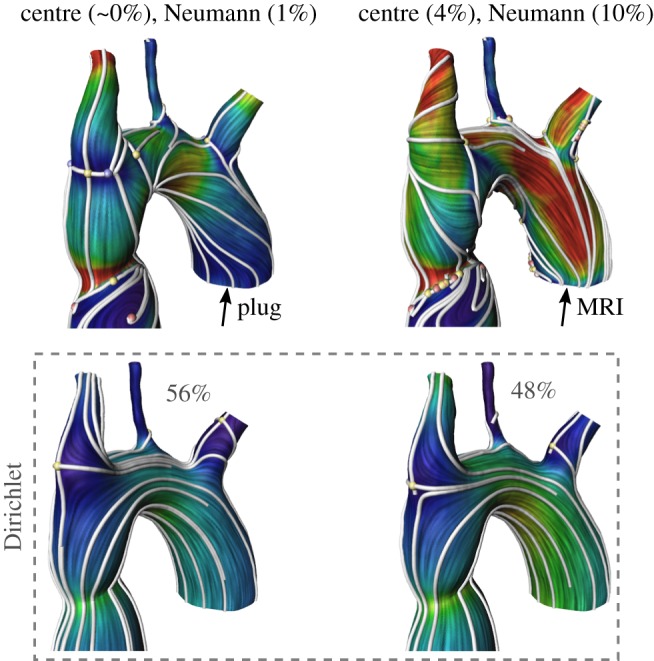
First row: Plug versus MRI input profile encoded in the centre and Neumann components. Second row: invariance of the Dirichlet component under the change of input profile.

**Figure 6.**
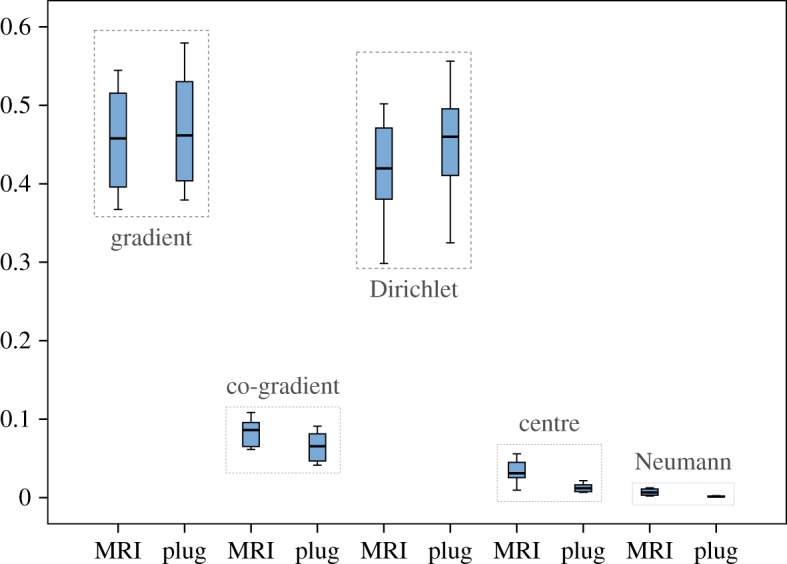
Comparison of the WSS of 10 healthy patients with MRI versus plug inlet velocity profile. The noise produced from the MRI can be identified by a significant increase of centre and Neumann fields.

The boundary conditions used in CFD are, most of the time, either a constant input velocity field (plug) or velocity information acquired from 4D MRI scans using specialized software and sequences. MRI profiles are noisy and sometimes the resulting WSS field looks more perturbed than a WSS field obtained by a plug profile. Using the HMF-decomposition, one can classify which components of the WSS are more affected by the inlet velocity profile. Figure 5 presents a WSS analysis of the same patient with a different inlet profile. Visually, the global flow pattern of the two vector fields are different, but by analysing each HMF-decomposition component, one can see perturbations in the centre and Neumann fields. The Dirichlet fields in both cases are topologically the same (similar streamline and same number of singularities). Figure 6 is a comparison of both inlet boundary conditions for 10 control patients. The statistical analysis (paired Student’s *t*-test) of normally distributed data (Komogorov–Smirnov test) found no significant difference for the gradient component (*p* = 0.571). However, significantly (*p* = 0.038) smaller Dirichlet components for MRI-measured inlet velocity profiles accompanied with significantly larger co-gradient (*p* = 0.007), centre (*p* = 0.002) and Neumann (*p* = 0.002) components of the HMF-decomposition have been observed. Notice that the *L*^2^-norms of the centre and Neumann components in both cases are relatively small compared with the other components.

### 3.4. Number of branches

**Figure 7.**
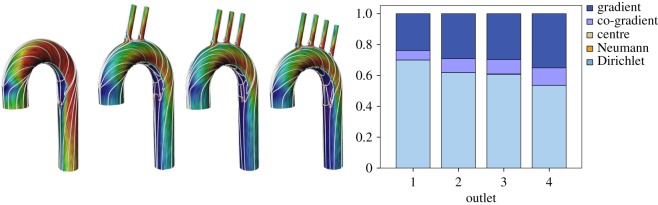
Analysis of artificial aorta models with a varying number of outlets. The streamlines show the flow of the gradient field. The diagram shows that the number of branching outlets is closely related to the gradient and Dirichlet field.

The following study shows the effect of branches on an idealized aorta. Starting with a curved cylinder with zero branch, artificial branches are successively added and a blood flow is simulated on each geometry using a plug inlet profile. The results show that for this ideal situation the gradient field increases with the number of branches (figure 7). Geometrically, branches induce high curvatures and hence more divergence. There are still several parameters not taken into account such as tapering or twisted cylinders. The proposed set-up with the correct geometry can be used to analyse these extra cases.

### 3.5. Unsteady flow

**Figure 8.**
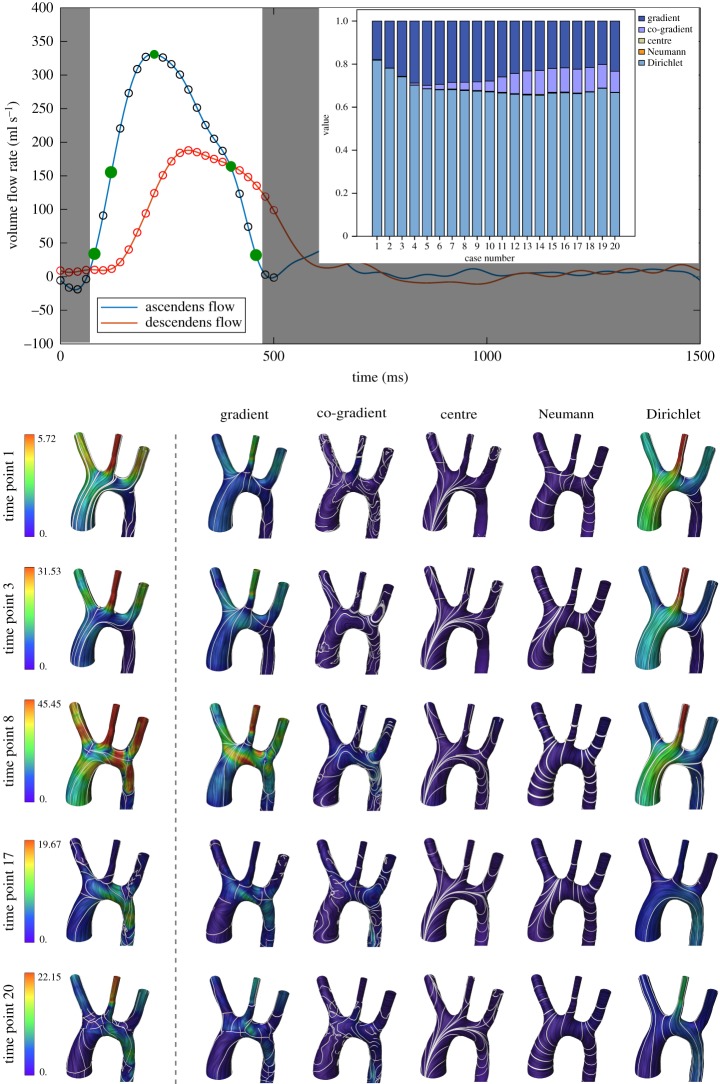
Evolution of the WSS HMF-decomposition in a CFD simulation with an unsteady flow. The diagram shows 20 time points of the simulation. The close-ups are five phases from the 20 time points of the unsteady flow simulation (green dots). The colours are relative to the min–max magnitude of each input vector field.

Finally, the effect of time-varying flow boundary conditions is examined in this section. For this purpose, an unsteady CFD simulation of a whole cardiac cycle performed earlier for a MICCAI CFD Challenge [25] was used. For the analysis, however, only the systolic part of the cardiac cycle is considered, since the diastolic part shows only little to zero flow and therefore negligible WSS in the aorta. Twenty time points have been evaluated in total and are presented in figure 8, along with inlet and outlet flow-curves. Additionally, decomposed WSS vector field plots are presented for five time points with a more detailed picture of the WSS distribution. As can be seen from the decomposition at the various time points, the respective components of the HMF-decomposition change over time, with a significant increase in co-gradient component and decrease in Dirichlet component. Furthermore, it appears that the variations of the HMF-components do not only arise from variations of the flow rate (i.e. Reynolds number) but also from acceleration and deceleration effects. This can be seen by comparing two time points with equivalent flow rates, as, for example, time points 3 and 17, both of which show a flow rate of about 160 ml s^−1^. Despite that, time point 17, at which the flow is being decelerated, shows a significantly higher co-gradient and lower gradient component than time point 3, where the flow is being accelerated. Neumann and centre components, however, remain at almost zero throughout the whole systole.

## 4. Method

**Figure 9.**
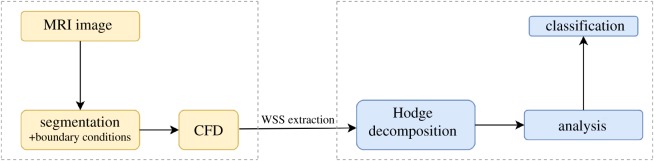
Analysis pipeline of WSS vector fields extracted from a simulated model and analysed via Hodge decomposition.

A diagram summarizing the analysis pipeline is given in figure 9. The implementation of the HMF-decomposition is done following the iterative *L*^2^-projection approach [20,22] with the discretization given in appendix A. The choice of basis functions for each harmonic field subspace follows [20]. Our system takes as input a mesh with a vector field and returns the five-term decomposition equation (2.1) of the vector field, assuming that the surface fulfils the topological requirement. Our implementation is done in Java using the JavaView (www.javaview.de) geometry processing package. The line integral convolution (LIC) implemented in ZIBAmira 2015.28 (Zuse Institute Berlin) is used for the field visualization. Maximum magnitude is coloured with red while close to zero vectors are coloured in violet. Most of the data used in this paper is from real patient biological models.

### 4.1. Data input

#### 4.1.1. MRI

The HMF-decomposition analysis was done for WSS vector fields of the aorta from an MRI-based CFD analysis of the aortic flow. These are subdivided in two groups: controls and coarctation of the aorta (CoA) patients before and after treatment.

The study was carried out according to the principles of the Declaration of Helsinki and approved by the local ethics committee. Written informed consent was obtained from the participants and/or their legal guardians.

MRI examinations used to set boundary conditions for the CFD analysis were performed using a 1.5 Tesla Achieva R5.1.8 MRI scanner with a five-element cardiac phased-array coil (Philips Medical Systems, Best, The Netherlands). MRI protocols including a routine three-dimensional anatomical imaging in end-diastole are used to reconstruct the geometry of the aorta (3D MRI). The sequence parameters used were: acquired voxel size 0.66 × 0.66 × 3.2 mm, reconstructed voxel size 0.66 × 0.66 × 1.6 mm, repetition time 4 ms, echo time 2 ms, flip angle 90°, number of signal averages 3. Four-dimensional velocity-encoded MRI (4D VEC MRI) was used to capture the flow data of the ascending aorta and the thoracic aorta (acquired voxel size 2.5 × 2.5 × 2.5 mm, reconstructed voxel size 1.7 × 1.7 × 2.5 mm, repetition time 3.5 ms, echo time 2.2 ms, flip angle 5°, 25 reconstructed cardiac phases, number of signal averages 1). High velocity encoding (3–6 m s^−1^) in all three directions was used in order to avoid phase wraps in the presence of valve stenosis or secondary flow. All flow measurements were completed with automatic correction of concomitant phase errors. These data were used to set inflow and outflow boundary conditions.

#### 4.1.2. CFD

**Figure 10.**
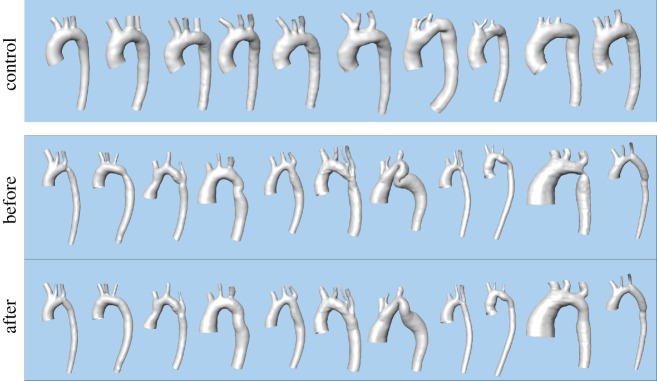
Segmented aorta reconstructed from MRI images and used for the CFD simulation.

CFD requires geometries. Geometries of human aortas were segmented and reconstructed using ZIBAmira 2015.28 (Zuse Institute Berlin, Berlin, Germany) according to the previous description [26]. Briefly, intensity-based image segmentation was done semi-automatically with an intense manual interaction. Rough surface geometries were then generated from segmentations with a subvoxel accuracy and subsequently smoothed using Meshmixer (v. 3.3, Autodesk, Inc., San Rafael, USA). These procedures were described in more detail earlier [26]. Figure 10 shows all aorta models used for our analysis.

With the exception of the unsteady case, all simulations were performed as steady-state simulations of the peak-systolic aortic flow using STAR-CCM+ (v. 12.06, Siemens PLM Software, Plano, USA). Vessel walls were assumed to be rigid and a no-slip boundary condition was applied at all walls. To model turbulence observed in systolic aortic haemodynamics, a *k* − *ω* SST turbulence model with a turbulence intensity of 5% at the velocity inlet was used. Blood was modelled as a non-Newtonian fluid with a constant density of 1050 kg m^−3^ and a Carreau–Yasuda viscosity model [27]. Patient-specific flow rates as measured with GTFlow (GyroTools LLC, Zurich, Switzerland) from 4D VEC MRI data were set at the LVOT inlet and the descending aorta outlet. Furthermore, patient-specific velocity profiles at peak-systolic flow rate were extracted using MEVISFlow (v. 10.3, Fraunhofer MEVIS, Bremen, Germany) and set as inlet boundary conditions. The used CFD pipeline was earlier validated by a comparison with 4D VEC MRI-measured velocity fields as well as clinically validated against catheter measured pressure drops in cases of CoA [28]. Furthermore, to validate results of our simulations we compare velocity fields calculated by CFD against velocity fields measured by 4D flow MRI, both visualized by velocity magnitude colour-coded path lines [29]. Calculated WSS values are in the range of published results [30].

### 4.2. Statistical analysis

Statistical analysis of the Hodge decomposition results was done using the software package IBM SPSS Statistic, version 25 (IBM, USA). Measured data are presented as mean and standard deviation (s.d.) for normally distributed data or as a median with IQR. All data were tested for normality using the Kolmogorov–Smirnov test. Depending on the results of the normality test, the Student’s *t*-test or Mann–Whitney *U*-test were used for the group comparison. Paired tests were used to compare pre- and post-treatment results. A *p*-value of less than 0.05 was considered significant.

## 5. Results

**Figure 11.**
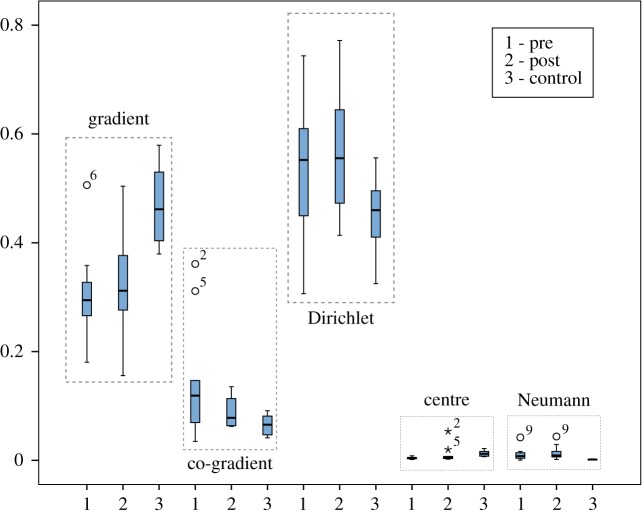
Comparison of the WSS of 11 patients before and after intervention, and 10 healthy patients. An improvement in gradient and Dirichlet together with a reduction in co-gradient is observed.

Results of the HMF-decomposition analysis of 11 CoA patients before and after treatment as well as 10 controls are illustrated in figure 11. The Student’s *t*-test found significantly lower gradient and significantly higher Dirichlet in CoA cases before treatment versus controls: 0.3 (s.d. = 0.083) versus 0.46 (s.d. = 0.065) gradient, and 0.54 (s.d. = 0.125) versus 0.42 (s.d. = 0.065) Dirichlet. The co-gradient in the CoA group was higher than in controls with median 0.119 IQR [0.069–0.147] versus median 0.086 IQR [0.065–0.098], approaching significance (Mann–Whitney test, *p* = 0.061). Overall significant reduction (paired Wilcoxon test, *p* = 0.041) in co-gradient has been observed from pre- (median 0.119 IQR [0.069–0.147]) to post-intervention (median 0.070 IQR [0.064–0.113]) as expected from the theoretical experimentation exposed previously. However, no significant changes in the major flow descriptors of gradient (*p* = 0.174) and Dirichlet (*p* = 0.073) were found between pre- and post-treatment WSS vector fields (paired Student’s *t*-test).

## 6. Discussion

Our first results on the application of a discrete HMF-decomposition analysis of the aortic flow and especially an analysis of the WSS vector fields of the coarctation of the aorta (congenital narrowing of the aorta) disease revealed great potential for computational biofluid mechanics. Based on the results shown in figure 11, we suppose that the HMF-decomposition analysis allows us to find anatomical shapes forming pathological haemodynamics before disease progress becomes symptomatic.

Our findings show an added value of the HMF-decomposition analysis if compared with the usually used analysis of WSS vector fields by visualization or quantification of time- and surface-averaged WSS values, areas with low WSS values (e.g. WSS values below 0.5 Pa) or areas with high OSI as well as an analysis of WSS critical points [6,12,30]. Our approach, however, does not allow, for example, a quantitative analysis of two different abnormal WSS vector fields or a quantitative analysis of different impact factors (boundary conditions) forming abnormal haemodynamics.

The results shown in figure 11 together with the theoretical analysis on ideal models raise several open questions. Could a pathological anomaly such us stenosis present in the aorta be identified by its amount of WSS co-gradient? Control healthy patients have less co-gradient field. The pre- versus post-operative patient also shows a significant reduction in co-gradient field. An objective classification has not been achieved with our current analysis because of the limited number of patient models. Nevertheless, the theoretical deformation shown in figure 4 suggests that it should generally be the case.

The current analysis is focusing only on studying the differences in WSS vector fields shown by the HMF-decomposition due to treatment aiming at restoring the stenosed region towards a physiological diameter. The differences between diseased and control groups aiming to identify haemodynamic reasons for the development of a pathological anatomy are emphasized. Future research could be also focused on the impact or decomposition of haemodynamic and/or morphometric boundary conditions on the resulting HMF WSS vector field decomposition. This is, however, a challenging task since the haemodynamics depend on a set of nonlinear effects of all boundary conditions including the flow rate distributions, vessel curvature, branching topology and others.

A perfect flow would have a pure WSS Dirichlet field, but due to branches and taperings in the shape, gradient and co-gradient components are also present. On the one hand, there are higher gradient than Dirichlet components in the control group; on the other hand, there are higher Dirichlet than gradient components in the pre- and post-operative groups. The theoretical analysis on the number of branches shows that the nature of the gradient field may change and become dominant. Understanding the correlation between the gradient and the Dirichlet field will be a good direction for future research.

We applied the HMF-decomposition first to analyse WSS vector fields, since WSS is a known risk factor for the genesis and progress of pathological processes associated with an interaction between blood flow and vessel wall. The analysis allows for an integral characterization of the WSS distribution. However, it does not replace an analysis of WSS magnitudes, which are also associated with abnormal blood flow conditions: regions with low WSS promote development of atherosclerosis and thrombus formations, whereas high WSS could cause an injury of endothelial cells. As part of our study, we investigated the impact of side branches, degree of stenosis and/or treatment procedure, inlet flow profile boundary conditions and the impact of laminar flow disturbances on WSS vector fields as characterized by the HMF-decomposition.

The HMF-decomposition analysis of simulated WSS vector fields was based on the Reynolds-averaged Navier–Stokes (RANS) solver using the *k* − *ω* SST turbulence model. However, flow simulations of haemodynamics allowing assessment of pressure and velocity fields and hence WSS are not limited to the RANS CFD. The lattice-Boltzman method (LBM), large-eddy simulations (LES) or smoothed-particle hydrodynamics (SPH) are possible CFD alternatives. For example, LES is supposed to be better suited in order to simulate accurately transition to turbulence and to assess turbulent structures [31]. Finally, the choice of the CFD approach should be done based on validation studies comparing simulation results versus *in vivo* measurements [32]. The HMF-decomposition analysis is, however, independent from the CFD approach.

Further possible and planned studies include, for example, an analysis of pulsatile flows, analysis of flow differences due to different turbulence models, the extension of an analysis to other parts of circulation (e.g. coronary arteries, carotid bifurcations or cerebral vessels) and other diseases (e.g. abdominal aortic aneurysms, cerebral aneurysms or coronary artery disease).

Summarizing our results, the HMF-decomposition is able to support (1) basic research of the flow-mediated disease, (2) predictive computational modelling of the treatment procedure as well as (3) quantitative analysis of the haemodynamic treatment outcome. Altogether, it supports a clinical translation of the computational modelling approach.

## 7. Conclusion

The novel discrete Hodge–Morrey–Friedrichs decomposition was for the first time applied to analyse the WSS vector fields of simulated patient-specific aortic blood flows. The approach seems to be a powerful tool to distinguish between pathological and physiologic blood flows, and to characterize the impact of inflow boundary conditions as well as the impact of a treatment.

## Figures and Tables

**Figure 12.**
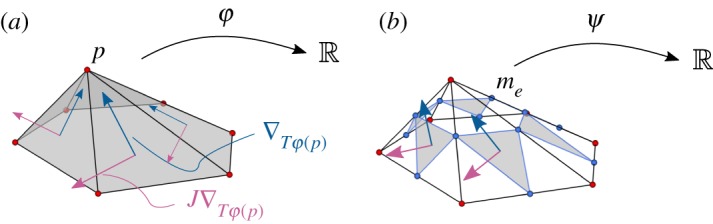
Examples of a function *φ* ∈ *S*_*h*_ together with ∇φ, J∇φ (*a*) and *ψ* ∈ *S*_h_^*^ with ∇ψ, and J∇ψ (*b*) defined over the triangle *T*.

**Figure 13.**
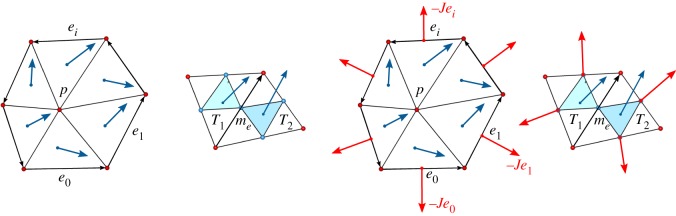
Computation of the curl and divergence of a vector field at a point on $\nabla S_{h}$ and $J\nabla S_{h}^{*}$.

## Data accessibility

Our data are deposited at https://datadryad.org/resource/doi:10.5061/dryad.mn46cd6 [33].

## Appendix A

In this appendix, we give a brief introduction to calculus on discrete surfaces. Only the most relevant notions necessary to understand the discrete Hodge decomposition are given. A complete overview can be found in [22].

**A.1. Simplicial surfaces**

A two-dimensional *simplicial surface* *M*_*h*_ is a set of triangles glued at their edges with a manifold structure. In finite-element analysis, this type of discrete surface is called *triangle mesh*. For actual FEM computations on such meshes, one often uses the space *S*_*h*_ of linear Lagrange functions, or the space $S_{h}^{*}$ of Crouzeix–Raviart functions. They are defined by

$$\begin{array}{rl} S_{h} & :=\{\varphi \;\;:\;\;M_{h}\to R\;|\;\varphi_{|T}\;\text{is linear on each triangle}\;T,\text{and globally continuous}\}\\ S_{h}^{*} & :=\{\psi \;\;:\;\;M_{h}\to R\;|\;\psi_{|T}\;\text{is linear},\;\text{and continuous at edge midpoints}\}. \end{array}$$

A geometric realization of example functions on *S*_*h*_ and $S_{h}^{*}$ is shown in figure 12. Two additional subspaces *S*_0_ ⊂ *S*_*h*_ and $S_{0}^{*}$ ⊂ $S_{h}^{*}$ for surfaces with boundary are given by

$$\begin{array}{rl} S_{0} & :=\{\varphi \in S_{h}\;|\;\varphi (v)=0\;\text{ for all boundary vertices}\;v\}\\ S_{0}^{*} & :=\{\psi \in S_{h}^{*}\;|\;\psi (m_{e})=0\;\text{for all boundary edge midpoints }\;m_{e}\}. \end{array}$$

The *gradient field* ∇φ of a function *φ* ∈ *S*_*h*_ or $S_{h}^{*}$ is a constant tangent vector in each triangle. The *co-gradient field* J∇φ is obtained by a rotation *J* of the gradient ∇φ by *π*/2 in each triangle (figure 12). The idea of having functional spaces is a common technique in finite-element analyses to solve complicated partial differential equations. For example, a temperature map *u* which assigns a scalar value to each vertex of *M*_*h*_ is an element of *S*_*h*_. It can be expressed with respect to the nodal basis functions (*φ*_*i*_)_*i*_ of *S*_*h*_, i.e. $u=\sum_{i}u_{i}\varphi_{i}$, where *φ*_*i*_ is the Kronecker delta, *φ*_*i*_(*v*_*j*_) = 1 if *i* = *j* and 0 otherwise, for a vertex *v*_*j*_ of *M*_*h*_. Then, the gradient of an arbitrary function in *S*_*h*_ can simply be expressed as a linear combination of the $\nabla \varphi_{i}$s.

**A.2. Vector fields on simplicial surfaces**

Definition A.1 (PCVF). The space of *piecewise constant tangential vector fields* *Λ*^1^(*M*_*h*_) on a two-dimensional simplicial surface $M_{h}\subset R^{n}$ is given by

$$\Lambda^{1}(M_{h}):=\{X:M_{h}\to TM_{h}\;|\;X_{|\mathrm{triangle}\;T}\;\text{is a constant tangent vector in}\;T\}.$$

Here, *TM*_*h*_ denotes the (piecewise) tangent bundle of *M*_*h*_. The gradient field ∇φ introduced previously is an example of a tangential vector field.

Definition A.2 (*L*^2^-product). The *L*^2^-*product* of two vector fields, $X={\left(X_{T}\right)}_{T\in M_{h}}$ and $Y={\left(Y_{T}\right)}_{T\in M_{h}}$, where $X_{T},Y_{T}$ are tangent vectors in the triangle *T*, is defined by the area-weighted Euclidean sum

$${\left\langle X,Y\right\rangle }_{L^{2}}=\sum_{T\in M_{h}}\langle X_{T},Y_{T}\rangle \text{Area}(T).$$

In particular, two vector fields X and $Y\in X_{h}$ are *L**^2^-orthogonal* if ${\left\langle X,Y\right\rangle }_{L^{2}}=0$. A vector field subspace *A* ⊆ *Λ*^1^(*M*_*h*_) is the *L**^2^-orthogonal decomposition* of two subspaces *B*, *C*⊆ *A* (written A=B⊕C) if every X∈A can be written uniquely as a sum X=Y+Z with Y∈B and Z∈C, and furthermore ⟨Y,Z⟩=0. The sum of two vector fields is a new vector field obtained by the sum of the components.

**A.3. Discrete calculus**

Definition A.3 (Discrete curl). The *discrete curl* of a vector field $X={\left(X_{T}\right)}_{T\in M_{h}}$ at a vertex *p* and an edge midpoint *m*_*e*_ of *M*_*h*_ is computed by

$$\begin{array}{rl} {\mathrm{curl}}_{h}X(p) & :=\frac{1}{2}\oint_{\partial \;\mathrm{star}\;p}X=\frac{1}{2}\sum_{i=1}^{k}\langle X_{|T_{i}},e_{i}\rangle \\ {\mathrm{curl}}_{h}^{*}X(m_{e}) & :=\oint_{\partial \;\mathrm{star}\;e}X=-\langle X_{|T_{1}},e\rangle +\langle X_{|T_{2}},e\rangle , \end{array}$$

where the *e*_*i*_s are the edges of the oriented boundary of star p, the *T*_*i*_s the triangles adjacent to *p* and *e* the edge with midpoint *m*_*e*_ (figure 13).

Definition A.4 (Discrete divergence). The *discrete divergence* of a vector field X at a vertex *p* and an edge midpoint *m*_*e*_ of *M*_*h*_ is computed by

$$\begin{array}{rl} {\mathrm{div}}_{h}X(p) & :=\frac{1}{2}\oint_{\partial \;\mathrm{star}\;p}\langle X,n\rangle \;\text{d}s=-\frac{1}{2}\sum_{i=1}^{k}\langle X_{|T_{i}},Je_{i}\rangle \\ {\mathrm{div}}_{h}^{*}X(m_{e}) & :=\oint_{\partial \;\mathrm{star}\;e}\langle X,n\rangle \;\text{d}s=\langle X_{|T_{1}},J_{|T_{1}}e\rangle +\langle X_{|T_{2}},J_{|T_{2}}e\rangle , \end{array}$$

where **n** is the outer unit normal along ∂ star p, respectively, ∂ star e. Discrete rotation and divergence are related by ${\mathrm{curl}}_{h}JX={\mathrm{div}}_{h}X$ and ${\mathrm{curl}}_{h}^{*}JX={\mathrm{div}}_{h}^{*}X$ (cf. figure 13).

Definition A.5 (Dirichlet and Neumann field). A vector field X is a *Dirichlet field* (respectively, *Neumann field*) if $X_{|T\in \partial M_{h}}$ is orthogonal (respectively, ‘almost’ parallel) to the boundary edge of *T*.

In the discrete case, the definition of Neumann fields is subtle due to technical properties of the chosen function spaces *S*_*h*_ and $S_{h}^{*}$. They are not strictly parallel along the boundary as one might expect from the smooth case, but can deviate slightly. However, they are overall mostly parallel, so for simplicity one may imagine them as being just parallel, in perfect duality to the definition of Dirichlet fields. For technical details, we refer the reader to [20, §3.1].

The harmonic Dirichlet field $H_{D}$ is, for example, a divergence-free and a curl-free field orthogonal to ∂*M*_*h*_. Note that Dirichlet fields and Neumann fields do not exist on a closed surface. One can nevertheless define them using hard directional constraints on certain features of the underlying surface, e.g. sharp features.
